# The impact of incorporating *Lactobacillus acidophilus* bacteriocin with inulin and FOS on yogurt quality

**DOI:** 10.1038/s41598-022-17633-x

**Published:** 2022-08-04

**Authors:** Heba Hussien, Hagar S. Abd-Rabou, Marwa A. Saad

**Affiliations:** 1 Department of Food Hygiene and Control, Faculty of Veterinary Medicine, University of Sadat City, Sadat City, 32897 Egypt; 2 Food Technology Department, Arid Lands Cultivation Research Institute, The City of Scientific Research and Technological Applications (SRTA-City), Alexandria, 21934 Egypt; 3 Food Hygiene and Control Department, Faculty of Veterinary Medicine, Menoufia University, Shebin Elkoum, 32514 Egypt

**Keywords:** Health care, Microbiology, Antimicrobials, Bacteria, Industrial microbiology

## Abstract

The current study aimed to figure out the effect of using a combination of 2% inulin, and 2% Fructo-oligosaccharides (FOS) with *Lactobacillus acidophilus* and their bacteriocin on some yogurt properties such as coagulation time, extending the shelf life of set yogurt and its microbiological quality, also the acceptance by consumers. The results indicated that coagulation time increased by 22.75% in yogurts prepared with *Lactobacillus acidophilus* and their bacteriocins compared to the control, and titratable acidity increased gradually in all treatments during storage. Hence control acidity (%) increased from 0.84 ± 0.02^A^ at zero time to 1.23 ± 0.03^A^ after 14 days of cold storage, while treatment (T4) was 0.72 ± 0.01^C^ at zero time and reached 1.20 ± 0.5^A^ after 39 days at the same conditions. The sensory properties showed the superiority of inulin, FOS, and *Lactobacillus* *acidophilus* bacteriocin groups. *Lactobacillus bulgaricus*, *Streptococcus thermophiles*, and *Lactobacillus acidophilus* count increased in the treatments compared to the control group, with an extended shelf life to 39 days of storage in the medicines containing *lactobacillus* *acidophilus* bacteriocin. Coliforms, Moulds, and yeasts did not detect in the treatments comprising 2% inulin, 2% FOS, and *lactobacillus* *acidophilus* bacteriocin for 39 days of refrigerated storage. This study proved that 2% inulin, 2% FOS, and *Lactobacillus* *acidophilus* bacteriocin fortification extended the shelf life by more than 5 weeks.

## Introduction

One of the most common commercial functional foods is fermented dairy products with probiotics, like milk drinks, yogurt, and cheese^1^. Yogurt is widely consumed for its therapeutically, nutritional, and sensory properties^2^, and it is considered the most usual vehicle for delivering functional ingredients^3^. Yogurt is obtained by fermentation whole, skimmed, or standardized milk through the action of *Lactobacillus delbrueckii* ssp. bulgaricus and Streptococcus thermophilus, which can be accompanied by other lactic acid bacteria that confer specific characteristics to the final product^4^. Probiotics have been defined as live microorganisms that, when administered adequately, consult a health benefit to the host^5^. Selecting a suitable base product for delivering probiotics is a crucial step toward developing probiotic foods^6^. Fermented milk has been used for a long time as the primary vehicle for probiotic strains.

Prebiotics were classified as selectively utilized substrates by host microorganisms conferring a health benefit^7^. Inulin and Fructooligosaccharides (FOS) are the most studied and well-established prebiotics. Inulin consists of a linear chain of fructose, constituted by a monomeric unit of fructose linked by β-(2, 1) glycoside bonds, with a terminal glucose unit linked by an-(1, 2) glycoside bond with a degree of polymerization (DP) more than 10. Fructooligosaccharide (FOS) is a fructose oligosaccharide linked by a β-(2, 1) glycosidic bond and terminated by a glucose molecule linked by α-(1, 2) glycosidic bond to fructose with DP < 10^8^. Prebiotic components can also supplement dairy products, such as cow milk yogurts^9,10^, resulting in improvements in the quality parameters of the products. Inulin has been described as improving dairy products’ texture^11^. Furthermore, other Fructo-oligosaccharides are fibers that have been employed together with probiotics in dairy products. Additionally, inulin-type fructans in yogurt may assist customers in consuming the recommended 25 g of dietary fiber daily from the European Food Safety Authority (EFSA). Additionally, consuming inulin has been linked to several health advantages, including a decreased risk of developing diabetes and obesity because of its impact on appetite and caloric intake through many mechanisms, including the synthesis of short-chain fatty acids (SCFA) due to colonic fermentation and the subsequent regulation of gut hormones^12^. Prebiotic components can also increase probiotic survival in probiotic dairy products, resulting in synbiotic outcomes^10^. The viability of bacteria is a vital characteristic of using probiotics in dairy products once they survive during the shelf life with minimum viable cells of 106–107 CFU mL^−1^ to provide health benefits to the host^13^. Control of pathogenic and spoilage microbes in various foods is crucial to ensuring the quality and safety of food. Bio-preservation has recently gained attention as a subject^14^; there is an urgent demand for a natural and safe method to boost the shelf life of fresh milk and dairy products; this technique is used to grow food shelf-life by applying a protective microbiota. The main probiotic bacteria that are commercially accessible are lactic acid bacteria (LAB) (*Lactobacilli*, *Streptococci*, *Lactococci*, and *Bifidobacteria*)^15^. They generate several metabolites, including organic acids, hydrogen peroxide, diacetyl, antifungal compounds, phenyl lactic acid, and bacteriocins, which guard the products against diseases and bacteria that can cause rotting^16–18^. In contrast to chemical preservatives, bacteriocins, produced by lactic acid bacteria (LAB), are widely used as safe additive preservatives, extending the shelf-life of food owing to their ability to inhibit a wide variety of pathogens^19^. Bacteriocins are antimicrobial peptides synthesized by lactic acid bacteria, extending the shelf-life and safety of food items^20^. Many species of the Lactobacillus genus are used to develop dairy products^21^. *Lactobacillus acidophilus* is a homofermentative, microaerophilic, short-chain, gram-positive bacteria with a rod morphology known as an effective bacteriocin-producing strain. Among the main products containing *Lactobacillus acidophilus*, La-5 are yogurt^22^ Ice cream^23^, cheese^24^, and dairy desserts^25^. The main objectives of this study were to evaluate the microbiological quality, viability of probiotic starter, physicochemical and sensory parameters of different yogurt formulations made using (inulin and FOS), and incorporation with lactobacillus acidophilus and its bacteriocin during 39-day refrigerated storage.

## Materials and methods

### Materials

Fresh raw cow and buffalo milk were obtained from the dairy selling outlet of the Faculty of Veterinary Medicine, Menofia University, Egypt, without any interaction with the herd. Lyophilized strains of *Lactobacillus acidophilus* DSMZ 20079, *Lactobacillus bulgaricus* (*Lb*. *bulgaricus*), and *Streptococcus thermophilus* were obtained from Cairo-MIRCEN (Microbiological Resource Center) Faculty of Agriculture, Ain Shams University, Cairo, Egypt. MRS broth (De Man, Rogosa, and Sharp) was obtained from Biolife, Italy. Inulin was obtained from Orafti^®^GR. Fructo-oligosaccharides (FOS) (Orafti^®^P95) were obtained from Belgium.

### Methods

#### Activation of lactobacillus strain and bacteriocin preparation

*Lactobacillus* (*Lb*) *acidophilus* DSMZ 20079 lyophilized pack was dissolved in MRS broth (de Man, Rogosa, and Sharp) at 37 °C for 24 h^26^ for activation. The activated culture was inoculated on MRS broth (1 L) aseptically and incubated at 37 °C for16 hrs. Then the culture was boiled in a water bath (WiseBath, Labortechnik, Germany) at 100 °C for 5 min to remove hydrogen peroxide. The cells were harvested by centrifugation at 10,000 rpm (Pro-Centrifuge, Centurion Science Limited, UK) for 20 min at 4 °C twice. The supernatants were collected, and the pH (Martini, Italy) was neutralized to (7) by (1 N) NaOH. The extract was then sterilized using a syringe filter of 0.45 µm pore size to obtain cell-free crude bacteriocin^27^.

#### Yogurt manufacture

Conventional lyophilized yogurt starter containing mixed cultures of *Lactobacillus bulgaricus* (*Lb. bulgaricus*) and *Streptococcus thermophilus* (1:1) were activated in sterile reconstituted skimmed milk powder (11%), and incubated at 37 °C for 24 h., then kept refrigerated to be used within in 24 h. Ten kilograms of skimmed milk were obtained by separating the milk fat using a separator and divided into two liters for five treatments; control, two probiotics, and two bacteriocins yogurt treatments supplemented with 2% inulin + 2% FOS, then mixed well^28,29^. The mixture was pasteurized at 85 °C for 30 min and immediately cooled to 45 °C, then inoculated with activated starter cultures (*Lb. bulgaricus* and *Streptococcus thermophilus*) then divided into the following treatments: [C: 2% yogurt starter cultures (Control)]; [T1: 1% yogurt starter cultures + 1% *Lb. acidophilus*]; [T2: 1% yogurt starter cultures + 1% *Lb. acidophilus* + 2% inulin + 2% FOS]; [T3: 1% yogurt starter cultures + 1% *Lb. acidophilus* bacteriocin]; [T4: 1% yogurt starter cultures + 1% *Lb. acidophilus* bacteriocin + 2% inulin + 2% FOS]. All treatments were mixed well and poured into sterile cups (100 ml), incubated at 42 °C until pH 4.6, then refrigerated at 4 °C.

#### Yogurt analysis

##### Coagulation time and Titratable acidity determination

The coagulation time for each yogurt group was calculated from the incubation's starting time until the curd's formation^30^. Titratable acidity (as % of lactic acid) was determined using the method described in^31^. The yogurt preparation and examination were repeated three times.

#### Microbiological analysis

Serial dilution was made to obtain the bacterial count^32^. *Lb. Acidophilus, Lb. bulgaricus*, and *Streptococcus thermophiles* were enumerated using the pouring plate method^33^. Enumerations of *Lb. Acidophilus* was carried out in MRS agar under the anaerobic condition at 37 °C for 48 h. *Lb. bulgaricus* was counted in MRS agar (pH 5.4) under aerobic incubation at 37 °C for 48 h., and *Streptococcus thermophilus* was counted on M17 agar at 42 °C for 48 h^34^. Sabouraud Dextrose agar medium supplemented with chloramphenicol (0.01%) was used to determine the total mold and yeast count after incubation at 25 °C for 5–7 days^35^.

#### Sensory analysis

The sensory evaluation of the yogurt samples was carried out according to^36^. The scores used were 60 points for flavor, 30 points for body and texture, and 10 points for appearance, with an overall score of 100.

#### Statistical analysis

Statistical analyses were performed using the one-way analysis of variance in SPSS 16.0. The results were considered significantly different, with p < 0.05 as^37^ described.

## Results and discussion

### Coagulation time and titratable acidity

Coagulation of milk results from the precipitation of milk protein (casein) in acidic conditions at a pH of around 4.6^38^. Table 1 shows that the treatment (T4) was prepared with 1% yogurt starter cultures 1:1 + 1% *Lb. acidophilus* bacteriocin + 2% inulin + 2% FOS has the longest coagulation time (4 h:10 min) with increasing percentage by 22.75% followed by treatment (T3) prepared by 1% yoghurt starter cultures 1:1 + 1% *Lb. acidophilus* bacteriocin (4 h.: 5 min) increasing percentage by 21.25%, than treatment (T2) prepared by 1% yoghurt starter cultures 1:1 + 1% *Lb. acidophilus* + 2% inulin + 2% FOS (3 h:50 min) increasing percentage by 4.79%. The lowest coagulation time was recorded for treatment (T1) which was prepared by 1% yogurt starter cultures 1:1 + 1% *Lb. Acidophilus* (3 h.:45 min) with an increasing percentage of 3.29% compared to the control treatment (coagulation time of control was 3.29). The variation in the coagulation time may be attributed to the antibacterial activity of probiotic bacteria and prebiotics (inulin and FOS) on the starter culture of yogurt and subsequent acid production^39^.

**Table 1:** Evaluation of the different yogurt treatments during cold storage: coagulation time, acidity, viability of starter bacteria, *Lactobacillus acidophilus*, and occurrence of yeast and molds.

Parameters	Days	C	T1	T2	T3	T4	Significance
Coagulation time per (hrs.: min)	Zero	3.34	3.45	3.50	4.05	4.10	
Increase (%)		0.00	3.29	4.79	21.25	22.75	
Acidity as lactic acid (%)	Zero	0.84 ± 0.02^A^	0.80 ± 0.04^A^	0.79 ± 0.01^B^	0.75 ± 0.03^B^	0.72 ± 0.01^C^	*
	7	0.95 ± 0.03^A^	0.97 ± 0.03^A^	0.95 ± 0.03^A^	0.83 ± 0.02^B^	0.79 ± 0.01^C^	*
	14	1.23 ± 0.03^A^	1.10 ± 0.02^B^	1.06 ± 0.05^BC^	0.90 ± 0.01^C^	0.85 ± 0.00^D^	*
	21	S	1.16 ± 0.03^A^	1.14 ± 0.02^A^	1.06 ± 0.03^B^	0.94 ± 0.02^C^	*
	28	S	S	1.22 ± 0.03^A^	1.17 ± 0.03^B^	1.08 ± 0.02^C^	*
	32	S	S	1.34 ± 0.03^A^	1.26 ± 0.05^B^	1.18 ± 0.3^C^	*
	39	S	S	S	S	1.20 ± 0.5^A^	
*Lactobacillus delbrueckii* sub spp. *bulgaricus* (log 10 cfu/g)	Zero	12.75 ± 0.15^A^	12.75 ± 0.03^A^	12.65 ± 0.05^B^	12.73 ± 0.07^A^	12.71 ± 0.02^A^	ns
	7	10.58 ± 0.04^D^	11.35 ± 0.01^C^	12.33 ± 0.05^B^	12.15 ± 0.11^B^	12.47 ± 0.09^A^	*
	14	9.00 ± 0.02^D^	10.40 ± 0.02^D^	11.86 ± 0.1^C^	11.53 ± 0.11^B^	12.31 ± 0.03^A^	*
	21	S	9.22 ± 0.03^D^	11.13 ± 0.09^B^	10.75 ± 0.06^C^	12.14 ± 0.05^A^	*
	28	S	S	10.35 ± 0.04^B^	10.52 ± 0.04^B^	11.24 ± 0.05^A^	*
	32	S	S	9.27 ± 0.12^C^	10.12 ± 0.03^B^	10.53 ± 0.11^A^	*
	39	S	S	S	S	9.52 ± 0.04^A^	
*Streptococcus thermophilus* (log 10 cfu/g)	Zero	9.77 ± 0.05^A^	9.54 ± 0.03^A^	9.76 ± 0.03^A^	9.62 ± 0.01^A^	9.73 ± 0.02^A^	ns
	7	8.50 ± 0.05^C^	9.11 ± 0.03^AB^	9.24 ± 0.01^B^	9.35 ± 0.02^B^	9.55 ± 0.01^A^	*
	14	7.16 ± 0.06^D^	8.16 ± 0.03^C^	9.01 ± 0.01^A^	8.43 ± 0.01^B^	9.00 ± 0.01^A^	*
	21	S	7.24 ± 0.05^C^	8.87 ± 0.03^A^	8.01 ± 0.03^B^	8.73 ± 0.03^A^	*
	28	S	S	7.98 ± 0.03^B^	7.68 ± 0.04^C^	8.15 ± 0.04^A^	*
	32	S	S	7.65 ± 0.04^B^	6.63 ± 0.04^C^	7.78 ± 0.04^A^	*
	39	S	S	S	S	7.72 ± 0.04^A^	
*Lactobacillus acidophilus* (log 10 cfu/g)	Zero		10.49 ± 0.11^A^	11.65 ± 0.08^A^			ns
	7		9.68 ± 0.06^B^	11.18 ± 0.06^AB^			*
	14		8.43 ± 0.06^C^	9.43 ± 0.18^B^			*
	21		7.54 ± 0.26^D^	8.84 ± 0.26^C^			*
	28		S	7.52 ± 0.26^D^			
	32		S	7.32 ± 0.26^D^			
	39		S	S			
Yeast and molds	Zero	ND	ND	ND	ND	ND	
	7	2.15 ± 0.01^B^	ND	ND	ND	ND	
	14	3.64 ± 0.04 ^A^	1.00 ± 0.01 ^B^	ND	ND	ND	*
	21	S	2.36 ± 0.03 ^A^	1.15 ± 0.02^C^	1.00 ± 0.05 ^C^	ND	*
	28	S	S	1.87 ± 0.01^B^	1.35 ± 0.05 ^B^	1.00 ± 0.03 ^C^	*
	32	S	S	2.16 ± 0.03^A^	2.14 ± 0.06 ^A^	1.85 ± 0.01^B^	*
	39	S	S	S	S	2.01 ± 0.05 ^C^	

C: 2% yogurt starter cultures 1:1 (control).

T1: 1% yogurt starter cultures 1:1 + 1% *Lb. acidophilus.*

T2: 1% yogurt starter cultures 1:1 + 1% *Lb. acidophilus* + 2% inulin + 2% FOS.

T3: 1% yogurt starter cultures 1:1 + 1% *Lb. acidophilus* bacteriocin.

T4: 1% yogurt starter cultures 1:1 + 1% *Lb. acidophilus* bacteriocin + 2% inulin + 2% FOS.

*The values indicated are the mean ± S.E (n = 3). Values in the same raw denoted by different letters (ABCD) differ significantly (p < 0.05) from each other.

*S* spoilage, *ND* not detected, ns non-significant.

During refrigerated storage (4 °C), the titratable acidity of yogurt samples in all treatment groups increased gradually as the storage period progressed (Table 1). The addition of inulin and (FOS) insignificantly affected the titratable acidity of yogurt samples. On the other hand, adding inulin and FOS increased the shelf life to 39 days in treatment T 4, which was prepared by 1% yogurt starter cultures 1:1 + 1% *Lb. acidophilus* bacteriocin + 2% inulin + 2% FOS compared to control treatment (C) which spoiled at 21 day of refrigerated storage. Treatment T2 prepared by 1% yoghurt starter cultures 1:1 + 1% *Lb. acidophilus* + 2% inulin + 2% FOS and T3: 1% yogurt starter cultures 1:1 + 1% *Lb. acidophilus* bacteriocin spoiled at 32 days of refrigerated storage. While treatment T1: 1% yoghurt starter cultures 1:1 + 1% *Lb. acidophilus* was spoiled at 28 days of refrigerated storage. EOSQ^40^ stated that the yogurt’s titratable acidity did not increase by 1.50%. Guven et al.^41^ found that yogurt's titratable acidity was not affected by the addition of different ratios of inulin, and it was increased during storage.

### Sensory evaluation

**Figure 1:**
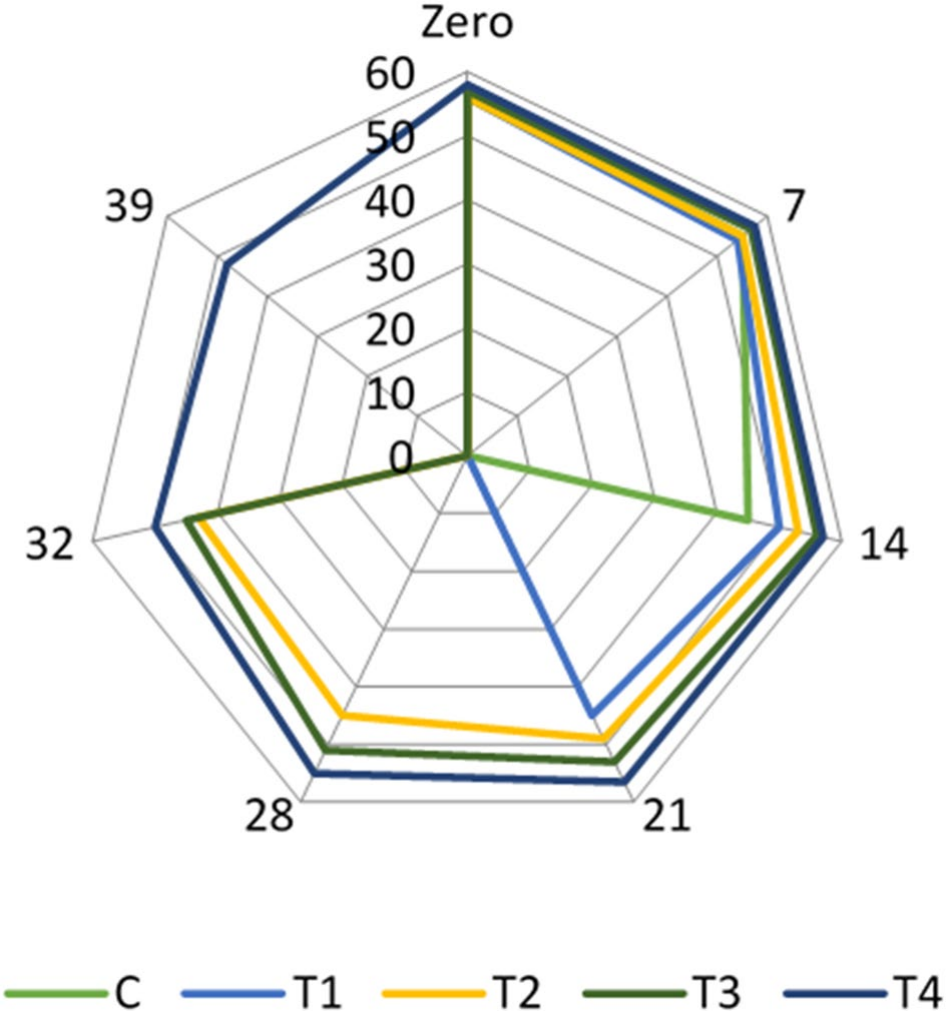
Flavour scores in the examined yogurt treatments throughout their refrigerated storage.

**Figure 2:**
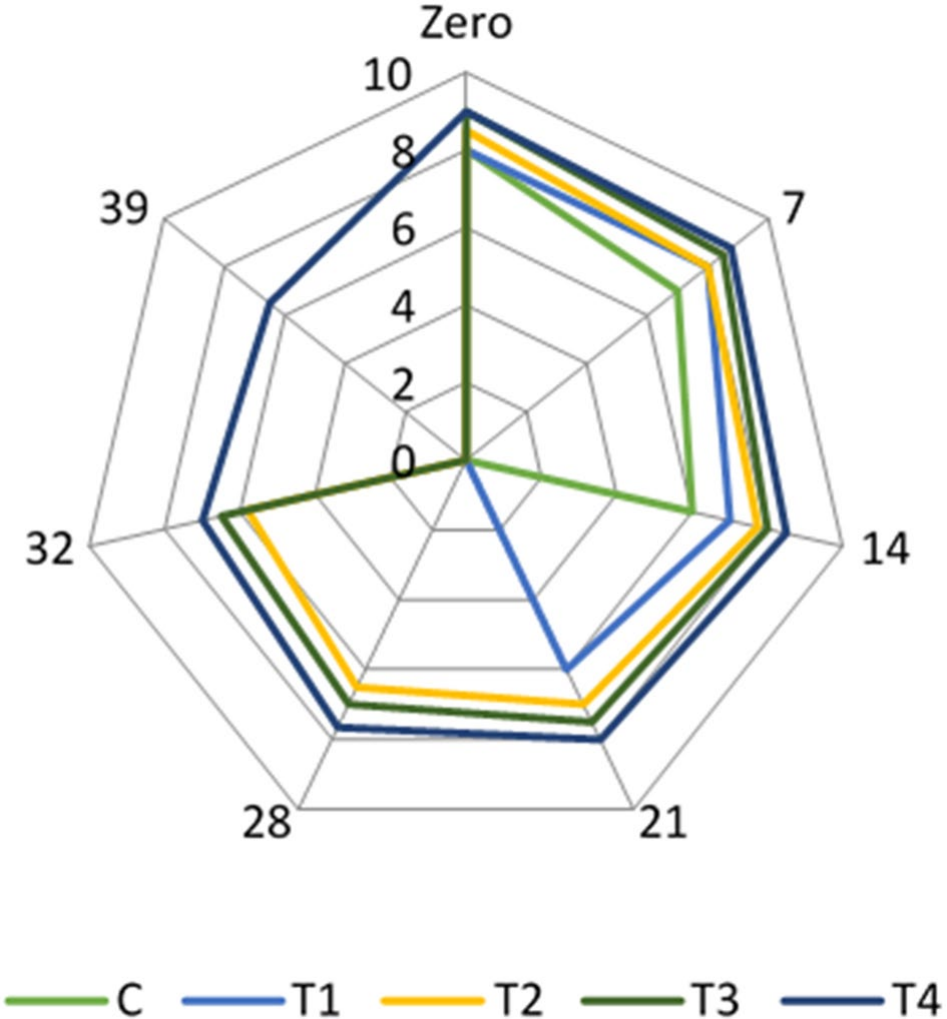
Appearance scores in the examined yogurt treatments throughout their refrigerated storage.

**Figure 3:**
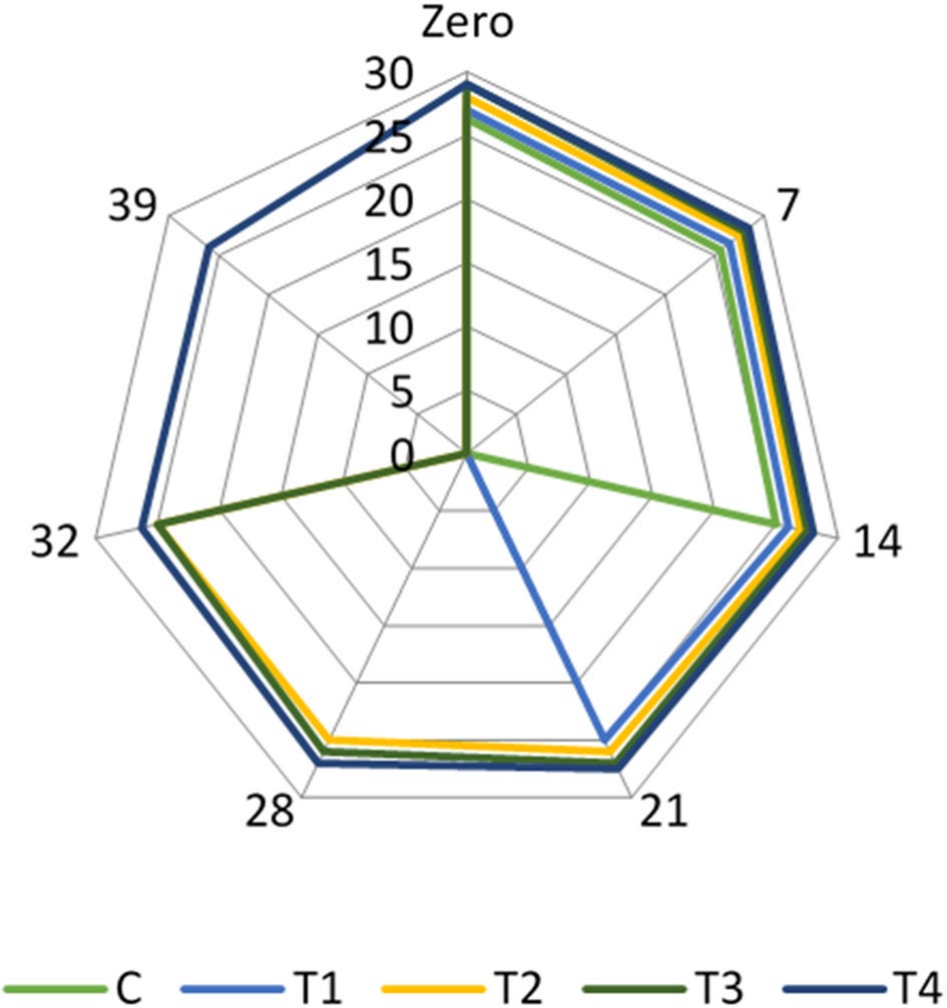
Body and Texture scores in the examined yogurt treatments throughout their refrigerated storage.

Adding inulin and FOS improved the sensory properties of the resultant yogurt samples. The result revealed that treatment (T2), 1% yogurt starter cultures 1:1 + 1% *Lb. acidophilus* + 2% inulin + 2% FOS and treatment (T4), 1% yoghurt starter cultures 1:1 + 1% *Lb. acidophilus* bacteriocin + 2% inulin + 2% FOS) were the highest accepted treatments regarding to sensory evaluation (Figs. 1, 2 and 3). Our results are in line with^42^, who reported that adding prebiotics could improve the physical and sensory properties of the yogurt. On the other hand, Cruz et al.^43^ explored that adding inulin may cause changes in yogurt quality attributes due to interactions between the functional ingredient and food matrix components. Inulin may provide yogurt mouth feel and sweet taste^44^.

Using inulin in yogurt production as carbohydrate fat substitutes can improve color perception^45^.

### Microbiological quality

Yogurt samples were examined microbiologically, as in table (1). There is a decline in the growth rate of *Lactobacillus delbrueckii* sub spp. *bulgaricus* in all examined samples. Treatments that contain inulin as in (T2): 1% yoghurt starter cultures 1:1 + 1% Lb. acidophilus + 2% inulin + 2% FOS and (T4): 1% yoghurt starter cultures 1:1 + 1% *Lb. acidophilus* bacteriocin + 2% inulin + 2% FOS have the highest count at the end of the storage period. The count of *Lactobacillus delbrueckii* sub spp. *bulgaricus* in treatment (T2) was 9.27 ± 0.12 CFU/g at 32 days of refrigerated storage, while for treatment (T4), the count was 9.52 ± 0.04 CFU/g at 39 days of refrigerated storage. Our result differs from that, who reported that *Lb. bulgaricus* in fermented skim milk prepared with 4% inulin showed a stable count during seven days of storage.

At the same time, *Streptococcus thermophiles* growth declined throughout the refrigerated storage. However, inulin and FOS groups have the highest count of *Streptococcus thermophiles*.

The count of *Streptococcus thermophiles* treatment (T2) was 7.65 ± 0.04 CFU/g at 32 days of refrigerated storage, and in treatment (T4), the count was 7.72 ± 0.04 CFU/g at 39 days of refrigerated storage (Table 1). These results agree with those obtained by^46,47^. They mentioned that inulin increases the growth and viability of lactic acid bacteria during fermentation or refrigerated storage. Furthermore, inulin concentrations were perfect for stimulating growth and retaining the viability of probiotic cultures in fermented milk^47,48^.

Table 1 shows the mean of *Lb. acidophilus* counts for the yogurt samples examined. *Lb. Acidophilus* counts declined throughout the storage period in T1 (yogurt with *Lb*. *acidophilus*) and T2 (yogurt with *Lb. acidophilus*, 2% inulin, and 2%FOS) with the final counts of *Lb. Acidophilus* was 7.54 ± 0.26 CFU/g on day 21 and 7.32 ± 0.26 CFU/g on day 32 and T2, respectively. The addition of inulin effectively increased the *Lb. Acidophilus* mean count when compared to the group that contains *Lb. Acidophilus* without inulin. These results agree with Donkor et al.^49–51^. They found that prebiotic ingredients such as inulin and FOS may exert a protective effect and improve the survival and activity of probiotic bacteria during the storage of probiotic food products.

The viability of probiotics was reported to be affected by many factors such as storage time, oxygen content, fluctuation in temperature, low pH, reduced water activity, and high concentration of salutes^52,53^.

Our study observed that adding inulin to probiotic yogurt enhances the growth of *S. thermophiles*, *L. bulgaricus,* and *L. acidophilus* until the end of the storage period compared with the treatments without inulin. The increase in probiotic counts of yogurt may be attributed to the action of inulin as a prebiotic substance. Akin et al.^54^ and^48,55^ attributed the increase in probiotic counts to the ability of probiotics and yogurt starter cultures to utilize inulin. Similarly, Donkor et al.^44^ showed that chicory-based inulin was a preferred carbon source for probiotic bacteria by increasing the growth performance and maintaining viability during cold storage. Acidity is one of the most critical factors that affect the viability of *S. thermophiles*, *L. bulgaricus,* and *L. acidophilus*^56^ as (FOS) act as substrates for the growth of LAB and inhibition of colonic cancer cells growth and putrefactive or pathogenic bacteria present in the colon through the production of short-chain fatty acids (SCFA). Rousseau et al.^57^ reported that FOS could stimulate the growth of the beneficial strains but not the pathogenic ones. The inhibitory effect against pathogenic bacteria usually results from the reduction in pH as a result of acid production, secretion of hydrogen peroxide, and release of natural antibiotics (bacteriocin) from beneficial microflora selectively stimulated by various prebiotics^58^.

In this study, (T4) yogurt samples appear to be the treatment that can resist spoilage with yeasts and molds (Table 1). According to EOSQ^40^, molds and yeasts count must not exceed ten cfu/g in yogurt. T4 was within a permissible limit until 28 days of storage with a mean count of 1.00 ± 0.03 CFU/g, while T2 was within the limit until 14 days of storage. T3 was within the allowable limit until 21 days of storage, with a mean count was 1.00 ± 0.05 CFU/g. T1 was within the permissible limit until 14 days of storage, with a mean count was 1.00 ± 0.01. At the same time, the control, yeast, and mold were not detected on zero-day only. These findings are consistent with the results obtained by Abee et al.^59^. They mentioned that LAB produces a proteinaceous antimicrobial substance known as bacteriocins that generally act through inactivation of enzymes, depolarization of the target cell membrane, or inhibition of the formation of the cell wall of pathogenic microflora including bacteria, mold, and yeast. Surajit et al.^60^ reported that inulin has antimicrobial activity against pathogenic microorganisms. Magnusson and Schnürer^61,62^ mentioned that lactic acid bacteria produced proteinaceous antifungal metabolites. *Lactobacillus acidophilus* has the most significant activity against *Aspergillus flavus* and *Aspergillus parasiticus*. In addition, *Lactobacillus acidophilus* has been shown to reduce aflatoxin production during 30 days of storage at room temperature in maize kernels^63^. The antifungal activity appears to be more assertive at lower pH ranges^62^; this may explain the antifungal activity of *Lb. Acidophilus* bacteriocin in acidic yogurt conditions. Batish et al.^63,64^ reported that *Lb. acidophilus* has antifungal activity. Coliform counts were not detected in any yogurt treatment on either the first day or during the storage period; this may be attributed to the good hygienic conditions during the preparation and storage of yogurts.

## Conclusion

This work studies the incorporation of inulin and FOS with (2%) as prebiotics with a traditional starter culture, including *Lactobacillus* *acidophilus* and its bacteriocin, to improve the quality of skim milk yogurt. This fortification increases the coagulation time compared with control in treatments containing *lb*. *acidophilus* bacteriocin and prebiotics mentioned above (T4) by 22.75%, which also has bacteriocin (T3). Inulin (2%) and FOS (2%) strongly stimulated the growth of *Lactobacillus acidophilus*, *Lactobacillus bulgaricus*, and *Streptococcus thermophilus*. *Lactobacillus acidophilus* and its bacteriocin with 2% inulin and 2% FOS gave the skim milk yogurt the best scores for flavor, body, texture, and appearance throughout refrigerated storage with an extended shelf life of up to 39 days and inhibit fungal growth without altering the development of the starter.

## Data Availability

The datasets used and/or analyzed during the current study are available from the corresponding author upon reasonable request.
